# Circulating soluble programmed death-1 levels may differentiate immune-tolerant phase from other phases and hepatocellular carcinoma from other clinical diseases in chronic hepatitis B virus infection

**DOI:** 10.18632/oncotarget.17546

**Published:** 2017-05-02

**Authors:** Na Li, Zhihua Zhou, Fang Li, Jiao Sang, Qunying Han, Yi Lv, Wenxuan Zhao, Chunyan Li, Zhengwen Liu

**Affiliations:** ^1^ Department of Infectious Diseases, First Affiliated Hospital of Xi'an Jiaotong University, Xi'an 710061, Shaanxi, China; ^2^ Department of Hepatobiliary Surgery, First Affiliated Hospital of Xi'an Jiaotong University, Xi'an 710061, Shaanxi, China; ^3^ Institute of Advanced Surgical Technology and Engineering, Xi'an Jiaotong University, Xi'an 710061, Shaanxi, China

**Keywords:** hepatocellular carcinoma, hepatitis B virus, soluble PD-1, infection phases, clinical diseases

## Abstract

Programmed death-1 (PD-1) is involved in the immune dysfunction of hepatitis B virus (HBV) infection and hepatocellular carcinoma (HCC). This study analyzed the association of circulating soluble PD-1 (sPD-1) levels with the phases and clinical diseases in chronic HBV infection. Serum sPD-1 levels were determined by enzyme linked immunosorbent assay in patients with different phases and liver diseases of chronic HBV infection. The sPD-1 levels in patients with chronic HBV infection were significantly elevated compared with HBV infection resolvers or healthy controls. According to phases, sPD-1 level in immune-tolerant phase (IT) was significantly lower than in other phases. Multivariate analysis showed that sPD-1 was an independent factor associated with IT. Area under the receiver operating characteristic (ROC) curves (AUC) showed that sPD-1 was significantly discriminative of IT from other phases with a cut-off of 1.535 ng/mL (AUC, 0.984; P<0.001). According to clinical diseases, sPD-1 level in HBV-related HCC was significantly higher than in other clinical diseases. Multivariate analysis showed that sPD-1 was an independent factor associated with HCC. The sPD-1 was significantly discriminative of HCC from other clinical diseases with a cut-off of 6.058 ng/mL (AUC, 0.962; P<0.001). The sPD-1 levels were significantly associated with HCC patients’ overall survival. HCC resection resulted in remarkable reduction in sPD-1 levels. These results demonstrate the involvement of sPD-1 in the disease course of chronic HBV infection and indicate the potential to apply sPD-1 as a biomarker for differentiating IT from other phases and HCC from other disease conditions in chronic HBV infection.

## INTRODUCTION

Hepatitis B virus (HBV) infection is a major global health problem with at least two billion people having been infected and approximately 240 million people being chronically infected with the virus [1, 2]. The Asia-Pacific region has a relatively high prevalence of HBV infection and patients with HBV infection in this region commonly acquire the infection either at birth or within the first 1-2 years of life, which is associated with a high incidence of chronicity [2, 3]. In China, the universal HBV vaccination in infants has reduced the prevalence of HBV infection, especially in children <5 years of age [4]. However, HBV infection and the associated liver diseases including hepatocellular carcinoma (HCC) remain a significant public health issue because of the existence of large number of individuals having been infected with the virus [5].

Chronic HBV infection is a dynamic process which may menifest several phases associated with various liver diseases depending on the interaction between the virus and the human host immune system [6]. The natural history of chronic HBV infection may be divided into five phases, namely, immune-tolerant phase (IT), immune-reactive phase (IR, also known as immune active/immune clearance/HBeAg-positive chronic hepatitis B/HBeAg clearance phase), low replicative phase (LR, also referred to as the “inactive HBsAg carrier” state), reactivation phase (RA, previously also referred to as “HBeAg-negative/anti-HBe positive chronic hepatitis B”), and HBsAg-negative phase (HN) [2, 6]. The virological, biochemical and pathological profiles and associated liver diseases in the phases may vary greatly and manifest differently. Briefly, IT is characterized by the active HBV replication with HBeAg positivity and high HBV DNA level in serum and the absence of abnormal biochemical liver function with no significant ongoing necroinflammatory disease of the liver; IR is characterized by the presence of HBeAg, high or fluctuating serum HBV DNA levels, persistent or intermittent elevation in serum aminotransferases, and active inflammation on liver biopsy; LR is characterized by absence of HBeAg, presence of anti-HBe, low or undetectable serum HBV DNA, persistently normal aminotransferase levels and mild inflammation and minimal fibrosis in the liver. Possible inactive cirrhosis may present in the liver in some cases; RA is characterized by negative or positive HBeAg, positive or negative anti-HBe, detectable HBV DNA, elevated aminotransferases, and continued necroinflammation in the liver; and HN is characterized by the loss of HBsAg, generally undetectable HBV DNA and detectable anti-HBc antibodies with or without anti-HBs although persistant low-level HBV replication with detectable HBV DNA in the liver may exist in some cases [2, 6]. Clinically, the liver diseases including asymptomatic chronic HBV carrier status (ACS), chronic hepatitis (CH), liver cirrhosis (LC) and HCC may develop in different phases during the natural history of chronic HBV infection although the incidence and severity of each disease condition may vary with the phases [6].

Immune dysregulation with T cell dysfunction in particular is a major characteristic of chronic HBV infection and contributes to the immunopathogenesis of HBV-associated liver diseases including the carcinogenesis of HBV-related HCC [7–13]. Programmed cell death-1 (PD-1), an inhibitory regulator of T cell activity, has been implicated in regulating immune responses to viral infections and tumors [14, 15]. Alteration in the expression and regulation of PD-1 has been demonstrated to be closely associated with T cell dysfunction in chronic HBV infection [8, 16–21] and HBV-related HCC [17, 19, 22–26], and blockade of PD-1 pathway has been shown to be able to restore T-cell responses in chronic HBV infection [8, 16, 20, 27]. Aside from the membrane bound form, PD-1 has a soluble form, soluble PD-1 (sPD-1). The sPD-1 is encoded by the alternative splice variant PD-1Deltaex3, which has a soluble extracellular domain but lacks the transmembrane domain of the PD-1 molecule [28]. The sPD-1 may functionally block the regulatory effect of membrane-bound PD-1 on T cells and lead to the alteration in T cell proliferation and regulation [29, 30]. It may also potently enhance antigen-specific T-cell immunity and dendritic cell maturation [31], rescue the proliferative response of virus-specific CD4 and CD8 T cells during chronic infection [32, 33], and facilitate antitumor immunity [34, 35].

Although the role of membrane bound PD-1 in T-cell dysfunction and exhaustion has been extensively studied, few studies have focused on evaluating the involvement of sPD-1 in chronic HBV infection and HBV-related liver diseases. One study indicated that sPD-1 is associated with sustained high HBV viral load and risk of HCC [36]. We, in this study, determined the circulating sPD-1 in patients with chronic HBV infection and analyzed the associations with the infection phases, liver diseases and the survival of patients with HBV-related HCC to further address the role of sPD-1 in the disease progression of chronic HBV infection and evaluate the potential value of sPD-1 determination in the differentiation of HBV infection phases and HBV-related liver diseases.

## RESULTS

### Demographic and clinical characteristics of the study subjects and sPD −1 levels

The 285 patients with chronic HBV infection included 213 males and 72 females aged 41.45 ± 13.85 (18-76) years, the 58 HBV infection resolvers included 39 males and 19 females aged 40.17 ± 11.84 (18-66) years, and the 86 healthy controls included 57 males and 29 females aged 39.26 ± 13.25 (19-74) years. The gender (M/F) and ages between the patients, resolvers and controls had no significant differences (Supplementary Table 1). The infection phases in the 285 patients with chronic HBV infection were categorized as 44 IT, 62 IR, 114 LR and 65 RA, and the clinical diagnoses of liver desease in the 285 patients were 44 ASC, 72 CH, 86 LC and 83 HCC (Supplementary Table 1).

The serum sPD-1 levels in patients with chronic HBV infection [3.80 (0.08-48.38) ng/mL] were significantly higher than in HBV infection resolvers [0.74 (0.09-16.56) ng/mL, P<0.001], and healthy controls [0.33 (0.04-5.15) ng/mL, P<0.001, Supplementary Figure 1]. The sPD-1 levels between HBV infection resolvers and healthy controls had no significant difference (P= 0.357, Supplementary Figure 1).

### sPD −1 levels according to HBV infection phases

**Figure 1:**
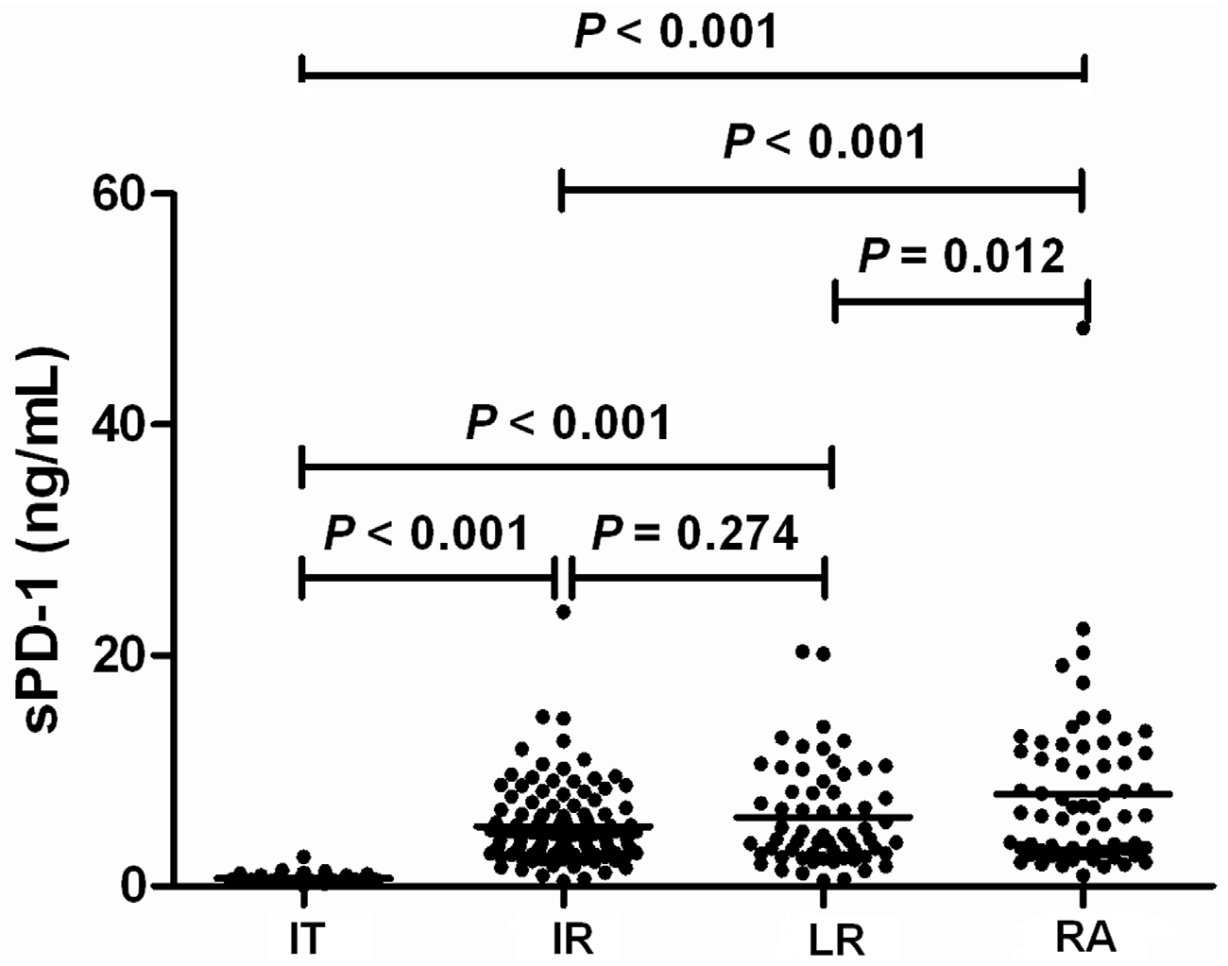
Serum sPD-1 levels according to the phases of chronic hepatitis B virus (HBV) infection IT, immune-tolerant phase; IR, immune-reactive phase; LR, low replicative phase; RA, reactivation phase.

The demographics and serum sPD-1 levels in patients at different phases of chronic HBV infection were shown in Table 1. The age, clinical diseases, HBV DNA levels, alanine aminotransferase (ALT) and aspartate aminotransferase (AST) levels, total bilirubin, and albumin were significantly different between IT, IR, LR, and RA (all *P* <0.001, Table 1). The serum sPD-1 levels among IT [0.62(0.08-2.54) ng/mL], IR[4.22 (0.45-20.32) ng/mL], LR [4.33 (0.42-23.76) ng/mL], and RA [6.13(0.93-48.38) ng/mL] were significantly different (P<0.001, Figure 1). The sPD-1 levels in IR, LR and RA were significantly higher than in IT (all P<0.001), the levels between IR and LR had no significant difference (P=0.274), the levels in RA were significantly higher than in LR (P=0.012) and in IR (P<0.001, Figure 1).

**Table 1:** Demographics and laboratory parameters in different phases of chronic HBV infection

	IT (n=44)	IR (n=62)	LR (n=114)	RA (n=65)	*P*
Gender (M/F)	29/15	47/15	81/33	56/9	0.067
Age [years, mean±SD (range)]	24.45±5.80 (18-49)	46.59±11.51 (22-72)	43.42±13.25 (18-76)	43.88±12.63 (18-64)	<0.001
Clinical diagnosis (ASC/CH/LC/HCC)	44/0/0/0	0/7/27/28	0/50/41/23	0/15/18/32	<0.001
HBV DNA (IU/mL, log)	7.45±1.07	3.61±0.84	6.45±1.24	5.09±1.43	<0.001
ALT (IU/L)	26.65 (12-38)	31 (7-84)	85 (14-1248)	95 (30-3629)	<0.001
AST (IU/L)	23 (13-92)	35 (15-201)	87 (22-1521)	104.5 (33-4082)	<0.001
Tbil (μmol/L)	13.01 (3.12-21.7)	17.97 (1.48-305.6)	28.02 (3.9-734.6)	33 (2-383.91)	<0.001
Albumin (g/L)	41 (36.9-50)	36.1 (18.8-50.7)	33.29 (20.9-49)	34.7 (21.1-46.76)	<0.001

IT: immune-tolerant phase; IR: immune-reactive phase; LR: low replicative phase; RA: reactivation phase; ASC: chronic asymptomatic HBV carrier; CH: chronic hepatitis; LC: liver cirrhosis; HCC: hepatocellular carcinoma; ALT: alanine aminotransferase; AST: aspartate aminotransferase; HBV: hepatitis B virus; Tbil: total bilirubin

Multivariate analysis using logistic regression showed that sPD-1 level was independently discriminative of IT from IR (P=0.003), LR (P=0.004), and RA (P=0.009, Table 2). The sPD-1 level was also independently discriminative of IT patients from all other non-IT patients (P=0.002, Table 2).

**Table 2:** Multivariate analysis using logistic regression for factors discriminating immune-tolerant phase from immune-reactive, low replicative and reactivation phases

Variable	IT *vs* IR (n=44) *vs* (n=62)			IT *vs* LR (n=44) *vs* (n=114)			IT *vs* RA (n=44) *vs* (n=65)			IT *vs* all non-IT (n=44) *vs* (n=241)		
	OR	SE	P	OR	SE	P	OR	SE	P	OR	SE	P
Gender	2.582	1.235	0.442	1.520	1.187	0.276	7.817	2.143	0.017	4.820	1.451	0.278
Age (years)	0.999	0.041	0.974	1.495	0.312	0.724	1.422	0.072	0.342	1.049	0.070	0.092
HBV DNA (IU/mL, log)	4.011	0.003	0.014	1.847	1.417	0.065	1.644	0.928	0.023	1.979	0.383	0.012
ALT (IU/L)	1.037	0.029	0.707	1.173	0.133	0.231	1.299	0.401	0.963	1.049	0.047	0.412
AST (IU/L)	0.987	0.022	0.537	1.238	0.151	0.157	1.458	0.265	0.964	1.039	0.036	0.040
Tbil (μmol/L)	1.026	0.040	0.519	0.927	0.052	0.149	1.841	0.291	0.694	0.988	0.019	0.521
Albumin (g/L)	0.939	0.067	0.345	0.587	0.421	0.206	1.251	0.971	0.896	1.793	0.150	0.122
sPD-1 (ng/mL)	0.532	0.212	0.003	0.233	0.680	0.004	0.225	0.971	0.009	0.263	0.054	0.002

IT: immune-tolerant phase; IR: immune-reactive phase; LR: low replicative phase; RA: reactivation phase; HBV: hepatitis B virus; ALT: alanine aminotransferase; AST: aspartate aminotransferase; Tbil: total bilirubin

**Figure 2:**
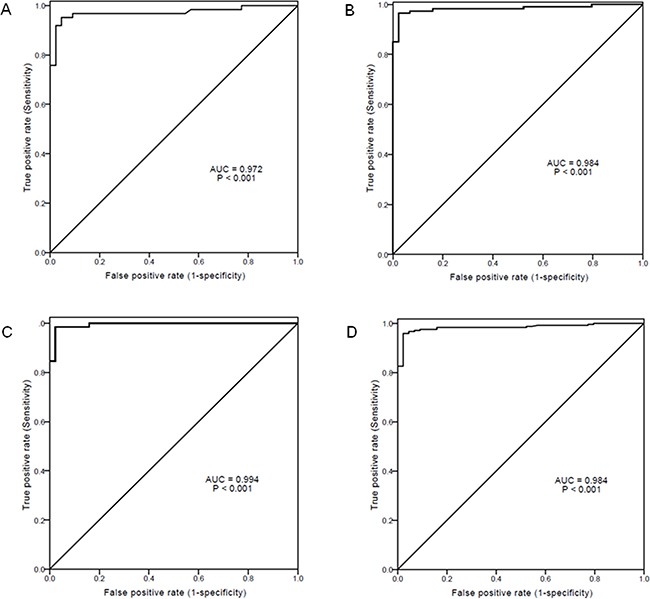
Receiver-operating characteristic (ROC) curves of serum sPD-1 levels for predicting immune-tolerant phase (IT) from other phases of chronic hepatitis B virus (HBV) infection **(A)** IT *vs* immune-reactive phase; **(B)** IT *vs* low replicative phase; **(C)** IT *vs* reactivation phase; **(D)** IT *vs* all other non-IT phases. AUC: area under ROC curve.

In view of the fact that IT patients had significantly different levels of sPD-1 in comparison with other infection phases, the ROC curves were plotted to evaluate the performance of serum sPD-1 in predicting IT versus other phases in chronic HBV infection. The area under ROC curve (AUC) value of sPD-1 levels was 0.972 for IT versus IR (P<0.001), 0.984 for IT versus LR (P<0.001), and 0.994 for IT versus RA (P<0.001, Figure 2). The AUC value of sPD-1 levels was 0.984 for IT versus all non-IT patients (P<0.001, Figure 2). With a cut-off value of 1.535 ng/mL in sPD-1 for IT versus all non-IT patients, the sensitivity and specificity were 0.985 and 0.977, respectively.

### sPD −1 levels according to clinical diseases of chronic HBV infection

**Figure 3:**
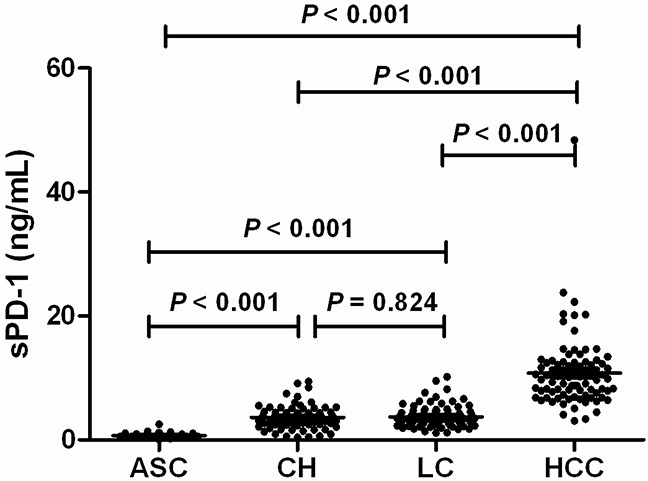
Serum sPD-1 levels according to the clinical diseases of chronic hepatitis B virus (HBV) infection ASC: chronic asymptomatic HBV carrier; CH: chronic hepatitis; LC: liver cirrhosis; HCC: hepatocellular carcinoma.

The demographics and laboratory parameters in patients with different clinical diseases of chronic HBV infection were shown in Table 3. The gender, age, HBV DNA levels, ALT and AST levels, total bilirubin, and albumin were significantly different between ASC, CH, LC, and HCC (all P<0.001 except P=0.002 in AST, Table 3). The serum sPD-1 levels among patients with ASC, CH, LC, and HCC were significantly different (P <0.001, Figure 3). The sPD-1 levels in patients with CH, LC, and HCC were significantly higher than in ASC (all P<0.001). The sPD-1 levels between CH and LC had no significant difference (P=0.824). The sPD-1 levels in HCC were significantly higher than in CH and LC (both P<0.001, Figure 3).

**Table 3:** Demographics and laboratory parameters in different clinical diseases of chronic HBV infection

	ASC (n=44)	CH (n=72)	LC (n=86)	HCC (n=83)	*P*
Gender (M/F)	29/15	50/22	58/28	76/7	<0.001
Age [years, mean±SD (range)]	24.45±5.80 (18-49)	34.42±11.66 (17-65)	46.69±11.00 (23-76)	50.56±9.79 (32-68)	<0.001
HBV DNA (IU/mL, log)	7.45±1.07	6.03±1.78	5.33±1.60	4.80±1.47	<0.001
ALT (IU/L)	26.65 (12-38)	168 (10-1248)	49.5 (9-504)	54 (7-3629)	<0.001
AST (IU/L)	23 (13-92)	104.5 (16.8-1521)	59 (19-474)	71(15-4082)	0.002
Tbil (μmol/L)	13.01 (3.12-21.7)	29.8 (2-734.6)	21.6 (4.8-244.3)	26.16 (1.48-727.24)	<0.001
Albumin (g/L)	41 (36.9-50)	36.65 (20.9-46.1)	30.85 (18.8-50.7)	33.4 (21.1-49)	<0.001

ASC: chronic asymptomatic HBV carrier; CH: chronic hepatitis; LC: liver cirrhosis; HCC: hepatocellular carcinoma; HBV: hepatitis B virus; ALT: alanine aminotransferase; AST: aspartate aminotransferase; Tbil: total bilirubin

Multivariate analysis using logistic regression showed that sPD-1 level was significantly discriminative of HCC from ASC (P=0.001), CH (P=0.005), and LC (P<0.001, Table 4). The sPD-1 level was also significantly discriminative of HCC patients from all other non-HCC patients (P<0.001, Table 4).

**Table 4:** Multivariate analysis using logistic regression for discriminating HCC from chronic asymptomatic HBV carrier, chronic hepatitis and liver cirrhosis

Variable	HCC *vs* ASC (n=83) *vs* (n=44)			HCC *vs* CH (n=83) *vs* (n=72)			HCC *vs* LC (n=83) *vs* (n=86)			HCC *vs* Patients without HCC (n=83) *vs* (n=202)		
	OR	SE	P	OR	SE	P	OR	SE	P	OR	SE	P
Gender	1.509	1.560	0.492	1.904	1.615	0.609	7.348	1.098	0.009	10.412	1.088	0.006
Age (years)	5.768	0.483	<0.001	1.495	0.153	0.009	1.041	0.033	0.221	1.081	0.028	0.005
HBV DNA (IU/mL, log)	0.260	0.340	<0.001	0.467	0.049	0.049	0.650	0.229	0.059	0.612	0.193	0.011
ALT (IU/L)	1.025	0.042	0.557	0.989	0.008	0.172	0.985	0.007	0.034	0.991	0.005	0.069
AST (IU/L)	1.024	0.030	0.433	1.006	0.007	0.338	1.012	0.006	0.056	1.006	0.004	0.130
Tbil (μmol/L)	1.133	0.125	0.150	1.101	0.005	0.047	1.004	0.009	0.660	0.997	0.003	0.352
Albumin (g/L)	0.798	0.122	0.065	0.861	0.097	0.120	1.107	0.053	0.056	1.067	0.047	0.165
sPD-1 (ng/mL)	13.698	0.397	0.001	9.497	0.945	0.005	3.050	0.198	<0.001	3.020	0.178	<0.001

ASC, chronic asymptomatic HBV carrier; CH, chronic hepatitis; LC, liver cirrhosis; HCC, hepatocellular carcinoma; HBV, hepatitis B virus; ALT, alanine aminotransferase; AST, aspartate aminotransferase; Tbil, total bilirubin.

**Figure 4:**
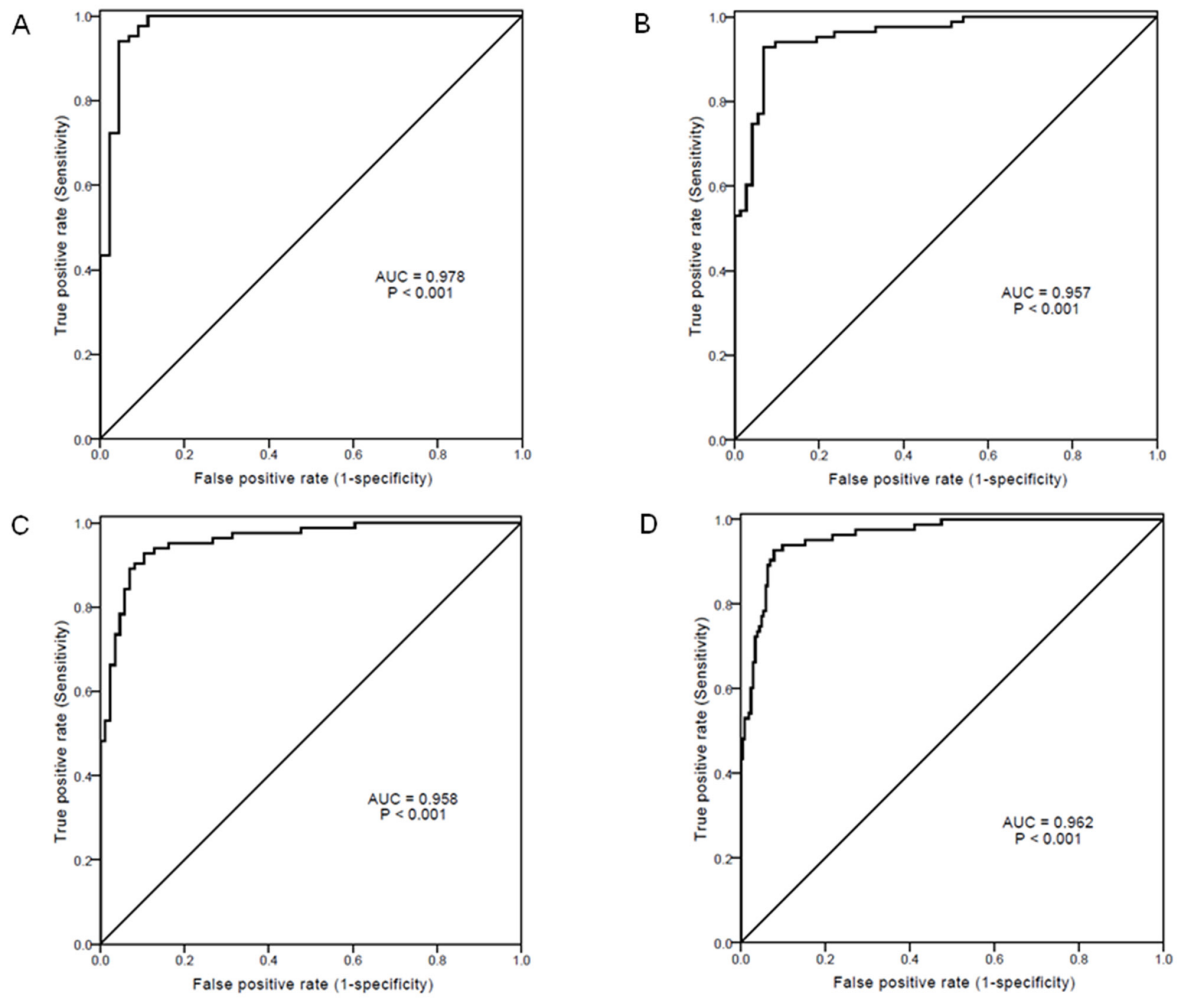
Receiver-operating characteristic (ROC) curves of serum sPD-1 levels for predicting hepatocellular carcinoma (HCC) from other clinical diseases **(A)** HCC *vs* chronic asymptomatic HBV carrier; **(B)** HCC *vs* chronic hepatitis; **(C)** HCC *vs* liver cirrhosis; **(D)** HCC *vs* all non-HCC. AUC, area under ROC curve.

In view of the fact that HCC patients had significantly increased levels of sPD-1 in comparison with other clinical diseases, the ROC curves were plotted to evaluate the performance of serum sPD-1 in predicting HCC versus other liver diseases in chronic HBV infection. The AUC value of sPD-1 levels was 0.978 for HCC versus ASC (P<0.001), 0.957 for HCC versus CH (P<0.001), and 0.958 for HCC versus LC (P<0.001, Figure 4). The AUC value of sPD-1 levels was 0.962 for HCC versus all non-HCC patients (P<0.001, Figure 4). With a cut-off value of 6.058 ng/mL in sPD-1 for HCC versus non-HCC, the sensitivity and specificity were 0.928 and 0.931, respectively.

### Association between sPD-1 and other clinical parameters

When the association between serum sPD-1 and other parameters was analyzed according to infection phases, no significant association was observed in IT and IR. In LR, sPD-1 level was significantly associated with ALT and AST levels (P=0.017 and P=0.013, respectively, Supplementary Table 2). In RA, PD-1 level was significantly associated with age (P=0.005, Supplementary Table 2). When the association between serum sPD-1 and other parameters was analyzed according to clinical diseases, no significant association was observed in ASC and CH. In LC patients, sPD-1 level was significantly associated with ALT and AST levels (P= 0.030 and P= 0.004, respectively, Supplementary Table 3). In HCC patients, sPD-1 level was significantly associated with AST levels (P=0.024, Supplementary Table 3). When all the patients with chronic HBV infection were analyzed, sPD-1 levels were significantly associated with HBV DNA levels and AST levels (P= 0.002 and P=0.047, respectively, Supplementary Table 3).

### Association of sPD-1 levels with overal survival of HCC patients

**Figure 5:**
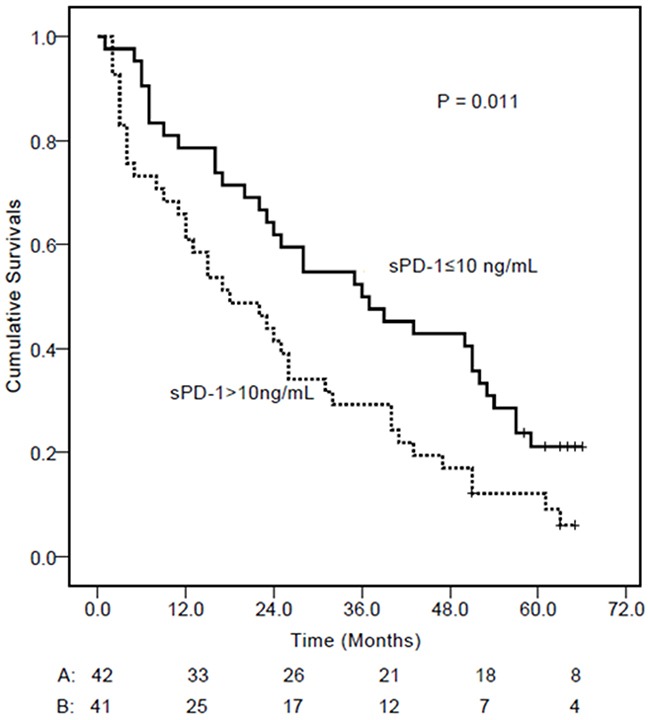
Kaplan-Meier curves of overal survival in patients with hepatitis B virus-associated hepatocellular carcinoma (HCC) according to serum sPD-1 levels At the cut-off value of 10 ng/mL identified by the receiver operating characteristic curve, HCC patients with a sPD-1 level ≤10 ng/mL had longer cumulative survival.

Univariate and multivariate analysis showed that, together with gender, platelet count, MELD score, tumor size, tumor nodules, and vascular invasion, sPD-1 level was a significantly independent factor associated with the overal survival of HCC patients [HR (95%CI)= 1.622 (1.068-2.694), P= 0.026, Table 5]. The Kaplan-Meier curves showed that the overal survival of patients with HBV-associated HCC with sPD-1 levels >10 ng/mL was significantly lower than those with sPD-1 levels ≤10 ng/mL (P= 0.011, Figure 5).

**Table 5:** Univariate and multivariate analysis of factors associated with the overall survival of hepatocellular carcinoma patients

	No. of patients	Overall survival (%)			Univariate analysis	Multivariate analysis	
		1 year	3 year	5 year	P	HR (95%CI)	P
Gender					0.873	10.794 (2.196-27.541)	0.002
Male	76	71.1	40.8	11.8			
Female	7	57.1	28.6	28.6			
Age(year)					0.360	1.001 (0.989-1.027)	0.897
≤55	54	68.5	42.6	9.3			
>55	29	72.4	34.5	20.6			
HBV DNA (IU/mL)					0.876	1.109(0.812-1.967)	0.607
≤10^4^	29	79.3	44.8	10.3			
>10^4^	54	64.8	37.0	14.8			
ALT (IU/L)					0.726	1.176(0.882-1.973)	0.581
≤40	28	75.0	35.7	17.9			
>40	55	67.3	41.8	10.9			
AST (IU/L)					0.127	1.207(0.701-1.984)	0.552
≤40	28	85.7	64.3	28.6			
>40	55	61.8	27.3	5.4			
TBIL (μmol/L)					<0.001	1.143(0.812-2.076)	0.399
≤40	60	80.0	50.0	16.7			
>40	23	43.5	13.0	4.3			
Albumin (g/L)					<0.001	1.172(0.841-2.231)	0.305
≤32	38	57.9	26.3	2.6			
>32	45	80.0	51.1	22.2			
AFP (ng/mL)					0.036	1.097(0.524-1.891)	0.427
≤200	48	79.2	50.0	12.5			
>200	35	57.1	25.7	14.3			
Platelet count (×10^9^/L)					0.013	1.174(1.021-2.056)	0.039
≤50	36	60.2	29.6	8.6			
>50	47	81.1	55.7	26.7			
MELD score					0.002	1.627(1.059-2.328)	0.017
≤20	49	89.9	59.6	21.9			
>20	34	56.2	16.7	4.8			
Child-pugh grade					<0.001	1.142(0.821-1.994)	0.408
A	40	95.0	67.5	20.0			
B+C	43	46.5	13.9	6.9			
Tumor size (cm)					<0.001	1.629(1.007-2.357)	0.031
≤5	43	86.1	55.8	20.9			
>5	40	48.8	22.5	5.0			
Tumor nodules					<0.001	1.213(1.007-1.873)	0.044
Solitary	13	86.2	63.1	29.9			
Multiple	47	52.8	27.4	5.7			
Diffuse	23	15.7	8.9	0			
TNM stage					<0.001	1.721(0.874-2.477)	0.061
I+II	57	91.2	57.9	19.3			
III	26	23.1	0	0			
Vascular invasion					<0.001	1.279(1.054-2.098)	0.045
Yes	40	57.3	23.9	10.6			
No	43	86.1	49.4	22.9			
Presence of cirrhosis					0.022	1.259 (0.971-1.995)	0.079
Yes	62	62.4	30.9	10.3			
No	21	74.8	50.1	20.5			
sPD-1 (ng/mL)					0.011	1.622 (1.068-2.694)	0.026
≤10	42	78.6	50.0	19.0			
>10	41	60.9	29.3	9.8			

HR: hazard ratio; 95% CI: 95% confidence interval; HBV: hepatitis B virus; ALT: alanine aminotransferase; AST: aspartate aminotransferase; Tbil: total bilirubin; AFP: alpha-fetoprotein.

### Effect of HCC resection on sPD-1 levels

**Figure 6:**
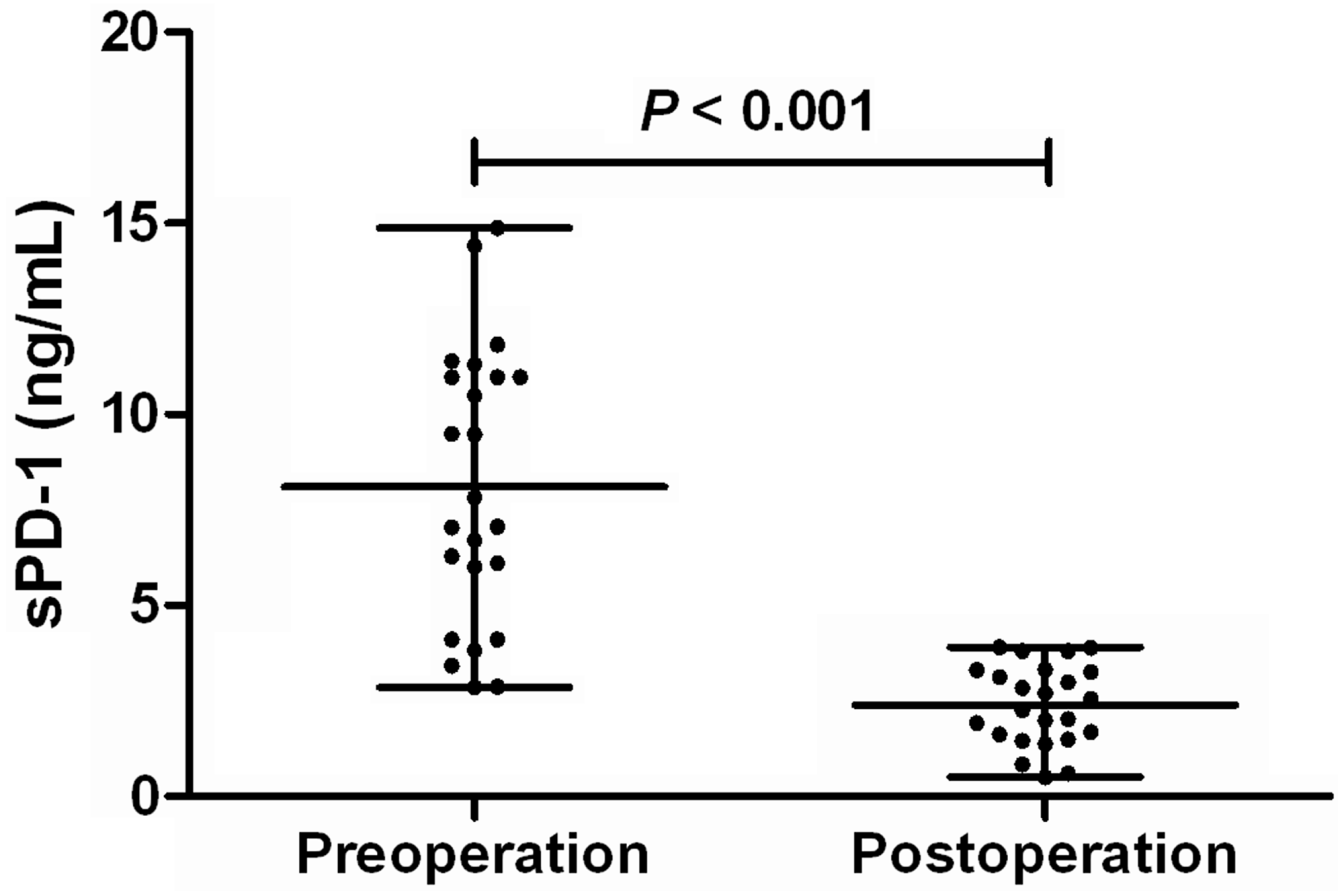
sPD-1 levels before and after tumor resection in patients with hepatitis B virus-associated hepatocellular carcinoma

In 24 patients with HBV-related HCC, serum samples both before and 2 days after the tumor resection were collected and the sPD-1 levels were determined. The sPD-1 levels were remarkbly reduced from 7.44 (2.86-14.87) ng/mL before the HCC resection to 2.41 (0.51-3.91) ng/mL 2 days after the resection of HCC (P< 0.001, Figure 6).

## DISCUSSION

The membrane bound PD-1 has been demonstrated to be complicated in the dysfunction of T-cell immune response to chronic HBV infection and the associated liver diseases including HCC by many studies [8, 16–20, 22–27]. However, few studies have evaluated the role of sPD-1 in chronic HBV infection and HBV-related liver diseases. Soluble PD-1 has been shown to potently enhance antigen-specific CD8+ T-cell responses and *in vivo* maturation of dendritic cells during activation of naive CD8+ T cells [31]. Excessive production of sPD-1 may counteract the function of overexpressed PD-1 to restrict inflammation [29, 30]. The sPD-1 has also been indicated to be functionally relevant to the immune response to chronic infection and tumor through its regulatory effect on membrane-bound PD-1 [29–32, 34]. Therefore, it is reasonable to assume the involvement of sPD-1 in chronic HBV infection and HBV-related liver diseases including HCC. In fact, a previous study has showed that sPD-1 is indicative of sustained high HBV viral load and risk of HCC [36]. Chronic HBV infection is a dynamic state of interactions between HBV and host immunity and may thus display different phases with various liver diseases [2, 6, 37]. Our results in this study showed that serum sPD-1 levels were significantly elevated in patients with chronic HBV infection and closely associated with the phases and clinical diseases of chronic HBV infection.

According to the phases of chronic HBV infection, we showed that the sPD-1 levels were continuously increased from IT, IR, LR, to RA, with RA having the highest levels of sPD-1. The sPD-1 levels in IR, LR and RA were significantly higher than in IT. Low sPD-1 level was independently discriminative of IT from IR, LR, and RA. The sPD-1 level was also independently discriminative of IT patients from all other non-IT patients. The AUC values of sPD-1 levels further indicated that sPD-1 level was predictive of IT from all other phases with high sensitivity and specificity. These results suggest that elevated sPD-1 level is indicative of the immune activation in chronic HBV infection and the measurement of sPD-1 level may be used as a biomarker of the breaking of immune tolerence in chronic HBV infection which is an important reference for the initiation of treatment using either interferon or nucleos(t)ide analogues [6] and a potential rationale for the design of cancer immunotherapy [38].

The treatment of immunotolerant patients with chronic HBV infection is perplexed by the low response to current agents, interferon or nucleos(t)ide analogues, and the existence of advanced histological lesions, high HBV replication, and increased risk of HCC in some cases who usually had older age, which are associated with histological severity and patient outcome [39, 40]. Whether the monitoring of serum sPD-1, in addition to age, ALT, HBV DNA, liver biopsy and/or non-invasive assessment of fibrosis [39], may add novel information for the optimal and individualized management of immunotolerant patients is an interesting issue to be addressed.

According to liver diseases, we showed that the sPD-1 levels were continuously increased from ASC, CH, LC, to HCC, with HCC having the highest levels of sPD-1 significantly distinct from other disease conditions. Multivariate analysis showed that sPD-1 was independently discriminative of HCC from other liver diseases including ASC, CH and LC. The AUC values of sPD-1 levels further indicated that sPD-1 level was significantly predictive of HCC from all other clinical diseases with high sensitivity and specificity. Furthermore, sPD-1 levels were significantly associated with the overal survival of patients with HBV-related HCC, and removal of HCC by resection resulted in remarkable reduction of sPD-1 levels in HCC patients. These results are consistent with the previous findings showing that sPD-1 is associated with risk of HCC [36] and suggest that increased sPD-1 is obviously involved in the immunopathogenesis of HBV-associated HCC. The determination of serum sPD-1 levels may thus be potentially used as a prognostic biomarker of HBV-associated HCC if the findings in this study will be confirmed in prospective studies and larger sample size of patients.

Soluble PD-1 is indicated to be able to enhance antigen-specific T-cell immunity and dendritic cell maturation [31], restore the proliferative response of virus-specific CD4 and CD8 T cells during chronic infection [32, 33], and promote antitumor immunity [34, 35]. Taken together with findings that HBV-related HCC patients had the highest sPD-1 levels in this study, it is suggested that the obviously increased sPD-1 is a reflection of the activation of immune response to both the HBV and the tumor.

The association analysis between serum sPD-1 levels and clinical parameters showed that in LR and LC, sPD-1 levels were significantly associated with ALT and AST levels. In HCC, sPD-1 levels were significantly associated with AST. When all the patients were included in association analysis, sPD-1 levels were significantly associated with HBV DNA and AST levels. These results are partly consistent with the previous report indicating a significant association between sPD-1 and sustained high hepatitis B viral load [36] and suggest that sPD-1 is also associated with liver necroinflammatory reaction. Interestingly, we also found that sPD-1 levels were significantly associated with the age in RA patients. Of note, patients in RA phases had higher proportion of HCC in our patients and they usually had an older age which is an independent factor associated with the development of HCC [40–42]. These results further indicate the involvement of sPD-1 in HBV-related HCC.

PD-1 was shown to be highly expressed in tumour-infiltrating lymphocytes (TIL) of the liver tissues in HBV-related HCC [22, 26] and the expression levels correlated with portal vein tumor thrombosis [22], indicating the potential of PD-1 expression in TIL of liver tissues to serve as a prognostic marker of HBV-related HCC. In this respect, whether serum sPD-1 may be used as a potential surrogate of the PD-1 expression in TIL of liver tissues needs to be investigated.

Targeting the immune checkpoint proteins including PD-1 has been increasingly used in the treatment of many cancers including HCC with promising findings [43, 44]. Predictive biomarkers are necessary to optimize patient selection and minimize risk of toxicities. Although programmed cell death ligand 1 (PD-L1) expression has been primarily examined, this testing is insufficient for patient selection in most malignancies and the prediction accuracy is low [45–47]. In addition, the determination of PD-L1 expression requires an invasive procedure to obtain tumor tissues. Therefore, based on the close connection of serum sPD-1 levels with HBV-related HCC, it, speculatively, holds promise to examine the potential of serum sPD-1 as a noninvasive predictive biomarker for the immunotherapy of HCC through targeting PD-1 pathway.

To accurately define the disease phases in chronic HBV infection and to precisely identify patients who may benefit from antiviral treatment and/or immunotherapy are important for the management of patients with chronic HBV infection and HBV-related HCC. To this end, our findings in the present study indicate that the determination of serum sPD-1 may be helpful. However, it should be noted that this study had relatively small sample size of patient population, especially the HBV-related HCC patients with both sPD-1 levels being examined before and after tumor resection, and it has not investigated the potential predictive value of sPD-1 in relation to the treatment responses to antiviral therapy or immunotherapy of diseases associated with chronic HBV infection. Therefore, further studies with large patient population including patients under antiviral treatment or immunotherapy are warranted to confirm and extend the findings in this study.

In conclusion, this study demonstrate that increased serum sPD-1 levels are associated with the immune activation and liver inflammation associated with HBV viral replication and the hepatocarcinogenesis in chronic HBV infection, suggesting the involvement of sPD-1 in the disease course of chronic HBV infection and the possibility of using sPD-1 as a biomarker of immune activation and HCC development.

## MATERIALS AND METHODS

### Patients

Two hundred eighty-five patients with chronic HBV infection, 58 HBV infection resolvers and 86 healthy controls were included in this study. Patients were recruited from hospitalized patients in the First Affiliated Hospital of Xi'an Jiaotong University. Chronic HBV infection was defined by the serological positivity for HBsAg, HBeAg or anti-HBe and anti-HBc for more than 6 months. The phases of chronic HBV infection were defined according to the literatures [6, 48]. Patients with chronic HBV infection, who had no signs and symptoms, with normal ALT and AST levels, normal imaging of ultrasonography and/or computerized tomography (CT)) of liver and spleen, and no other evidence of liver diseases, were defined as asymptomatic HBV chronic carriers. Patients with chronic HBV infection, who had signs and symptoms of hepatitis and persistently abnormal liver function (elevated ALT, AST, and/or bilirubin) for more than 6 months, were diagnosed as with chronic hepatitis. The diagnosis of HBV-related liver cirrhosis was based on pathological cirrhosis of liver biopsy or imaging features of cirrhosis in ultrasound and CT or magnetic resonance imaging (MRI) in combination with HBsAg, HBeAg or anti-HBe, and anti-HBc seropositivity for more than six months, and abnormal liver function, portal hypertension with esophageal varices, splenomegaly, and ascites. The diagnosis of HBV-related HCC was based on pathological examination of surgical liver tissues in combination with angiography, ultrasound, CT, and MRI, and HBsAg, HBeAg or anti-HBe, and anti-HBc seropositivity for more than six months [49]. The infection resolvers were those who had regular physical examination performed and were tested positive for anti-HBs antibody and anti-HBc antibody, with normal liver function and liver imaging and with no history of hepatisis. The healthy controls were those who voluntarily donated blood, had no history of hepatitis and other diseases and were tested negative for serum markers of HBV infection. The patients with chronic HBV infection received no treatments before the samples were taken. Patients’ demographic and clinical data were collected. The HCC patients were followed-up for a median of 36 (1-77) months and they had a median overal survival of 25 (1-66) months. In an additional 24 patients with HBV-related HCC [18 males and 6 females, aged 47.92±13.86 (21-75) years], serum samples before and 2 days after tumor resection were collected for the determination of sPD-1. This study was performed in accordance with the Declaration of Helsinki and Ethics approval was obtained from the Ethics Committee of the First Affiliated Hospital of Xi'an Jiaotong University. All participants gave written informed consent.

### Determination of serum sPD-1 levels

Blood was collected intravenously from each participant after overnight fasting and cryopreserved at -20°C until use. Serum sPD-1 levels were quantitatively determined by using commercially available enzyme linked immunosorbent assay kit (Cloud-Clone Corp. 1304 Langham Creek Dr, Suite 226, Houston, TX). The intra-assay and inter-assay coefficients of variance (CV) for sPD-1 were <10% and <12%, respectively.

### Statistical analysis

Statistical analysis was performed by SPSS software version 16.0 (SPSS, Inc., Chicago, IL). Data were expressed as the mean ± SD or median (range). Continuous and categorical variables were compared between groups using the Mann-Whitney U test or Kruskall–Wallis rank-sum test. Multivariate analysis using logistic regression was performed for factors discriminating phases or clinical diseases. Correlation between serum sPD-1 levels and other parameters were analyzed using Spearman's rank tests. The receiver operating characteristic (ROC) curves were constructed and the area under curve (AUC) was computed to evaluate the performances of sPD-1 for discrminating IT or HCC from other infection phases or liver diseases. Univariate and multivariate analysis of factors associated with the overall survival of HCC patients was performed. Kaplan-Meier curves of overal survival in patients with HBV-associated HCC were plotted according to the serum sPD-1 levels. *P* values < 0.05 were considered statistically significant.

## SUPPLEMENTARY FIGURE AND TABLES


