# Supplementary material for: Circulating soluble programmed death-1 levels may differentiate immune-tolerant phase from other phases and hepatocellular carcinoma from other clinical diseases in chronic hepatitis B virus infection
Source: Oncotarget. 2017 May 2;8(28):46020–33. doi: 10.18632/oncotarget.17546 (PMC5542245; doi:10.18632/oncotarget.17546)
Supplement: Supplementary file 1 [file oncotarget-08-46020-s001.pdf]

Circulating soluble programmed death-1 levels may differentiate immune-tolerant phase from other phases and hepatocellular carcinoma from other clinical diseases in chronic hepatitis B virus infection

SUPPLEMENTARY MATERIALS

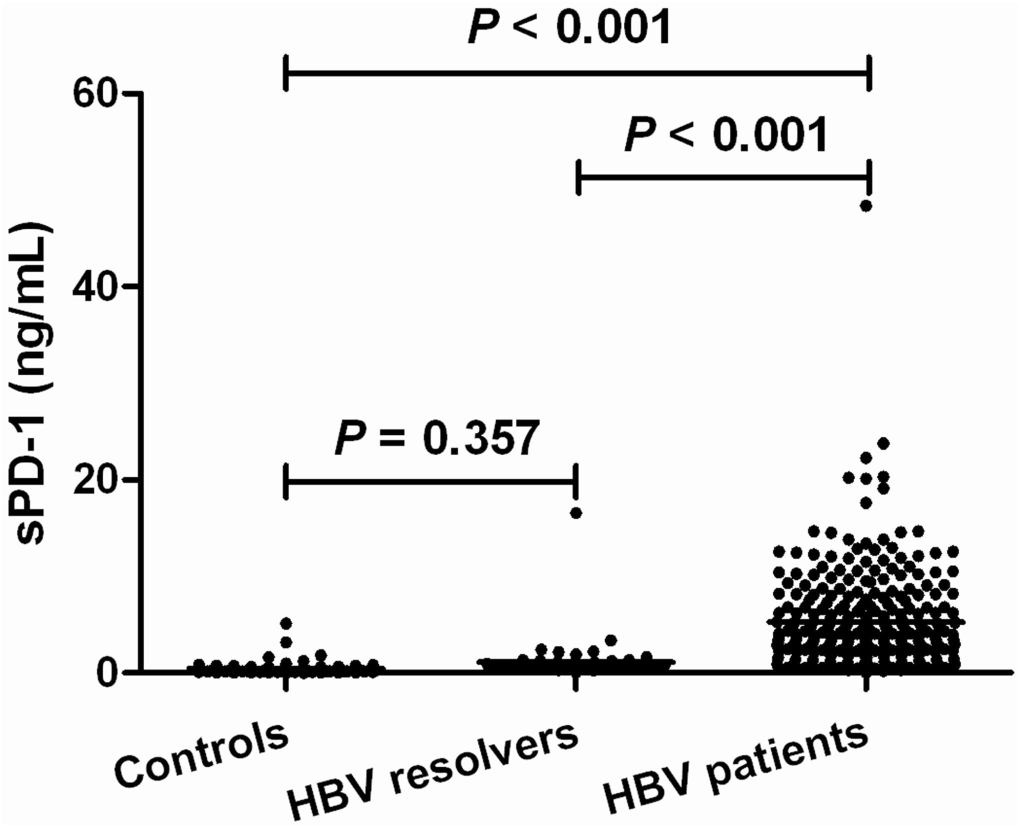

Supplementary Figure 1: Serum sPD-1 levels in hepatitis B virus (HBV) infected patients, HBV infection resolvers and healthy controls.

Supplementary Table 1: Demographics and serum PD-1 levels in patients with chronic hepatitis B virus (HBV) infection, HBV infection resolvers and healthy controls, and infection phases and clinical diagnoses in the patients

	HBV patients (n = 285)	HBV resolvers (n = 58)	Healthy controls (n = 86)	*P*
Gender (M/F)	213/72	39/19	57/29	0.211
Age [years, mean±SD (range)]	41.45 ± 13.85 (18-76)	40.17 ± 11.84(18-66)	39.26 ± 13.25 (19-74)	0.446
Infection phases				
IT	44			
IR	62			
LR	114			
RA	65			
Clinical diagnoses				
ASC	44			
CH	72			
LC	86			
HCC	83			

IT, immune-tolerant phase; IR, immune-reactive phase; LR, low replicative phase; RA, reactivation phase; ASC, chronic asymptomatic HBV carrier; CH, chronic hepatitis; LC, liver cirrhosis; HCC, hepatocellular carcinoma.

Supplementary Table 2: Correlation of serum sPD-1 levels with other parameters according to the phases of chronic HBV infection

Group (n)	Parameter	*r*	*P*
IT (n= 44)	Gender (M/F)	-0.200	0.193
	Age	0.199	0.194
	HBV DNA	0.115	0.457
	ALT	0.034	0.829
	AST	0.071	0.645
	Tbil	0.152	0.325
	Albumin	-0.052	0.737
IR (n=63)	Gender (M/F)	-0.095	0.464
	Age	0.179	0.164
	HBV DNA	0.101	0.435
	ALT	-0.170	0.187
	AST	0.027	0.835
	Tbil	0.175	0.173
	Albumin	-0.125	0.332
LR (n=114)	Gender (M/F)	0.019	0.843
	Age	-0.014	0.880
	HBV DNA	0.057	0.549
	ALT	0.223	0.017
	AST	0.231	0.013
	Tbil	0.036	0.706
	Albumin	-0.109	0.249
RA (n=65)	Gender (M/F)	-0.215	0.086
	Age	0.344	0.005
	HBV DNA	0.202	0.107
	ALT	0.043	0.731
	AST	0.099	0.435
	Tbil	0.197	0.116
	Albumin	-0.001	0.994

ALT, alanine aminotransferase; AST, aspartate aminotransferase; HBV, hepatitis B virus; Tbil, total bilirubin; IT, immune-tolerant phase; IR, immune-reactive phase; LR, low replicative phase; RA, reactivation phase.

Supplementary Table 3: Correlation of serum PD-1 levels with other parameters according to clinical diseases of chronic HBV infection

Group (n)	Parameter	*r*	*P*
ASC (n=44)	Gender (M/F)	-0.200	0.193
	Age	0.199	0.194
	HBV DNA	0.115	0.457
	ALT	0.034	0.829
	AST	0.071	0.645
	Tbil	0.152	0.325
	Albumin	-0.052	0.737
CH (n=72)	Gender (M/F)	-0.216	0.068
	Age	0.091	0.446
	HBV DNA	0.107	0.369
	ALT	0.178	0.134
	AST	0.183	0.124
	Tbil	0.204	0.085
	Albumin	-0.111	0.351
LC (n=87)	Gender (M/F)	0.031	0.056
	Age	0.207	0.056
	HBV DNA	0.136	0.213
	ALT	0.234	0.030
	AST	0.307	0.004
	Tbil	0.152	0.162
	Albumin	-0.068	0.536
HCC (n=83)	Gender (M/F)	0.121	0.275
	Age	0.026	0.815
	HBV DNA	0.021	0.851
	ALT	0.186	0.093
	AST	0.247	0.024
	Tbil	0.148	0.182
	Albumin	-0.133	0.232
	AFP	0.065	0.559
All (286)	Gender (M/F)	-0.103	0.083
	Age	0.393	0.726
	HBV DNA	0.232	0.002
	ALT	0.392	0.535
	AST	0.479	0.047
	Tbil	0.388	0.127
	Albumin	-0.331	0.253

ALT, alanine aminotransferase; AST, aspartate aminotransferase; HBV, hepatitis B virus; Tbil, total bilirubin; AFP, alpha-fetoprotein; ASC, chronic asymptomatic HBV carrier; CH, chronic hepatitis; LC, liver cirrhosis; HCC, hepatocellular carcinoma;
